# The Supra Renal Capsules

**Published:** 1858-03

**Authors:** 


### EDITORIAL DEPARTMENT.

---

*The Supra-Renal Capsules.*—Medical practitioners have always occasionally met with a disease affecting the color of the skin and accompanied by extreme emaciation, of the pathology of which they were utterly ignorant. We ourselves had an opportunity of seeing such a case in the wards of Prof. Flint, at the Buffalo Hospital of the Sisters of Charity. In this case, as far as our memory serves us, the patient was exceedingly emaciated; had cough and extensive pulmonary disease, and was affected with a brown discoloration of the skin, which was general, with the exception of spots over the body of dazzling whiteness, which were of irregular shape and and varied in size from that of a sixpence to a dollar, or in a few places were even larger. The patient died a few days after his admission into the wards, but no autopsy was made.

A short time after this, we saw a notice of a monograph by Dr. Addison, on "*The supra-renal capsules and their connection with Bronzed Skin.*" Dr. Addison had made a number of post-mortem examinations of patients who were affected with this peculiar discoloration of the integument, which he denominated "*Bronzed Skin,*" and invariably found associated with it a disease or disorganization of the supra-renal capsules, by which their function was entirely abolished. As Addison was the first to point out any connection between bronzed skin and disease of these organs, it has been proposed that the disease shall be called "Addison's Disease," as albuminuria connected with organic disease of the kidneys was named after its distinguished discoverer, Dr. Bright. Physiologists, as is well known, have never been able to determine definitely the function of the ductless glands or the blood glands, as they are sometimes called; which are the spleen, thynoid and thymus glands, mesenteric glands and the supra-renal capsules. These organs have all the anatomical characteristics of ordinary glands, with the exception of ducts; and, as owing to this, their secretion, if they have any, cannot be subjected to analysis, their *rôle* in the economy has remained almost unknown. In none of these however, has the obscurity been so great as in the case of the supra-renal capsules. The brilliant experiments of Bernard in regard to the Glucogenic function of the liver, which so surprised and delighted the scientific world, and which made such an advance into the pathology of that hitherto unaccountable disease, diabetes mellitus; gave a new impulse to the study of the ductless or blood glands; as by these experiments Bernard had shown that the liver, in addition to its office as a bile secreting organ, formed sugar within its substance, which was given up immediately to the mass of blood without the intervention of an excretory duct: since this was discovered, it has been shown that the liver also produces fat *de novo*, though as much is not known about this function as about the production of sugar. Thus the liver, in addition to being an ordinary gland, possessed of an excretory duct, is in reality a blood gland like the spleen, or supra-renal capsules.

A great proportion of the energy of the investigations into the functions of the ductless glands has been directed towards the spleen, and it is now supposed, and on pretty good foundation, that the spleen and mesenteric glands have an important office in the formation of blood globules. The physiology of the supra-renal capsules however, had still remained buried in its original obscurity.

Upon the appearance of the excellent monograph by Dr. Addison, observers in all parts of the world set themselves to work upon the physiology of the supra-renal capsules; the paper of Addison having thrown some light on the subject, and seeming indeed to establish some connexion between these organs and the formation of pigment, or the regulation of its deposition. The Physiologists who have made known to the world the results of their experiments, are Gratiolet, Brown Séquard, Philipeaux, and Dr. Harley, of University College, London. The experiments of these gentlemen, whose names our readers will recognise as belonging to men eminent in this department, have led them to somewhat different results: we had the pleasure of listening to lectures by Dr. Brown Séquard on this subject a year ago, when he seemed to prove that the supra renal capsules were essential in the highest degree to life, even more so than the kidneys themselves; as an animal survived for two or three days after the extirpation of the kidneys, but it lived for a few hours only after the removal of the supra-renal capsules. Brown Séquard also showed that animals from whom these organs had been removed, were convulsed and affected with that peculiar nervous phenomenon which is known as "turning," this turning always being in the same direction. He also asserts that death supervenes too rapidly to be due to inflammation of the peritoneum, and indeed, on examining this membrane after death, no evidences of inflammation are observed. Brown Séquard performed experiments in the presence of his audience, and we had an opportunity of witnessing the phenomena which he described.

The most elaborate and satisfactory paper on this subject which we have yet seen, is from the pen of Dr. Harley, and appeared in the last number of the British and Foreign Medico-Chirurgical Review. This article we have carefully perused, and it was this which led us to make the preceding observations. Thinking this subject to be of great importance to us as Physiologists and Pathologists, we have been thus extended in our remarks, and will give a brief recapitulation of the more important results at which Dr. Harley has arrived; this we deem the more appropriate, as it is probable that many of our readers do not have an opportunity of seeing the above mentioned periodical.

In anatomical structure, these organs present no very striking points of difference from some of the ordinary secreting glands of the economy; they are formed of a medullary and cortical substance like the kidneys. There is a peculiarity however in their chemical constitution. "In the beginning of the last year, M. Vulpian communicated to the *Societié de Biologie* the discovery of a very peculiar reaction possessed by the supra-renal capsules of vertebrated animals. He found that an aqueous solution of iodine brought in contact with the medullary substance gave rise to a beautiful rose color. And further, that the greater portion of oxidizing agents acted like the iodine solution, although in a minor degree. Even the oxygen of the air, under the influence of light, produced the same effect. I have repeated M. Vulpian's experiment on the medullary substance of the supra-renal capsules of sheep with success; but am equally at a loss with the author to form an opinion as to the nature of the substance which possesses the strange property alluded to. We as yet know so little regarding the nature of color and coloring matter, that it would be futile to attempt to draw any conclusions with regard to the import of the rose colored reaction above spoken of."

"Not being able to separate the coloring matter, M. Vulpian, in concert with M. Cloez, proceeded to extract the "immediate principles" of the supra-renal bodies; and these gentlemen have recently communicated to the *Institut de France* as the result of their united labors, the discovery of hippuric and tanrocholic acids in the supra-renal capsules of herbiverous animals. But as these substances have been abundantly found in different parts of the animal body, their discovery in the latter organs, although interesting, unfortunately throws no light upon the nature of their functions."

Dr. Harley has since seen a communication from Prof. Virchow, in which he confirms the observation of M. Vulpian with regard to the rose colored reaction, and also mentions that he has found senecine in the medullary substance.

The question arises whether the supra-renal capsules are organs peculiarly essential to fœtal existence and development. It was formerly supposed that this was the case, and the arguments in favor of this idea, were the great relative size of these bodies in the fœtus, and their small size in adults. Meckel states that at the third month, the supra-renal capsules and kidneys are of equal size, at the sixth month as 2 to 5, at birth as 1 to 8, and in adult life as 1 to 28. Later observations however, by the author, Brown Séquard, and others, show a different result in the case of some of the mamnalia. Dr. Harley has been confined in his observations to the cat, having examined thirteen fœtal and six adult capsules. Without giving the figures and calculations, we will give the results at which Dr. Harley has arrived and in which he confirms Ecker, Frey, and others: "The weight of the supra-renal capsule in the kitten at birth is to that of the kidney as 1 to 57.3; and in the adult as 1 to 54.8. But as so few adult capsules and kidneys were employed, it may be perhaps better to take the average of the whole six that were examined, which would then give us the relative weight of three organs in the full grown animal as 1 to 46.49.

"We shall find by a very simple calculation that the kidney was after birth increased 12.35 times in weight, while the supra-renal capsule at the same time has increased 14.87 times in weight. What conclusion are we to draw from this fact? We here see that the supra-renal capsules increase in size, as age advances, at a greater rate than the kidneys. I must here speak with caution however. Small statistics are dangerous data to draw conclusions from, and perhaps a larger number of observations might furnish us with different results."

Thus our readers will see that Ecker, Frey, and Brown Séquard, whose observations are here confirmed by Harley, have developed an exceedingly important fact in relation to the probable comparative activity of the function of the supra renal capsule, whatever it may be, in fœtal and adult life: correcting the doctrines promulgated by Meckel, that these organs in the mamualia diminish after birth, both in relative size and activity of function.

We now come to experiments, bearing directly on the function of the supra-renal capsules. The first experiment was on an adult cat, from which the right capsule was removed. The animal survived nine days after the operation, and on post mortem examination, no cause of death could be discovered.

Another experiment was made on a white cat, from which the capsules were removed. This died in twenty-four hours, and on examination there were found all the indications of peritonitis.

Another experiment was made on a small white coach dog, from which the left capsule was removed. It died on the twelfth day, and on examination, signs of peritonitis were discovered.

An experiment was then made on two Guinea pigs, in one of which the abdomen was opened, and the amount of injury which the parts would suffer by the removal of the supra-renal capsules, was inflicted, the wound sewed up, and the capsules allowed to remain; the fellow, which was of the same age, sex, and development, was then deprived of the capsule on the corresponding side. Both these animals died within twenty-four hours after the operation. The same experiment was then repeated on two cats. The cat from which the supra-renal capsule (the right,) was removed, lived two days; the other died during the third night. In the abdomens of both, signs of peritonitis were distinctly visible.

The next experiment was made on a bull terrier dog, by removing one of the capsules without applying a ligature to the vessels, the body being eunucleated with the fingers. The dog remained in the author's possession for five months, and did not suffer from a day's illness.

The next experiment was one of exceeding interest: it was on a large tom cat, in which, on opening the abdomen, the right capsule was rough and hard as a stone: it was euncleated with the greatest facility. The same operation was repeated on the other side with a similar result. On making a section of the capsules, it was found that a considerable portion of the medullary, as well as of the cortical substance, had become replaced by a calcareous deposit, consisting chiefly of carbonate of lime. The remaining portions of the gland contained so much fibrous tissue, that the normal structure might be said to have entirely disappeared. As the operation on this animal had been performed with the utmost facility, and there had been no hæmorrhage, or any apparent injury done to the surrounding parts, except the tearing through the vessels and nerves, the author felt sanguine of of success. His astonishment was great therefore when, arriving at the college next morning, he found the cat dead. The post mortem examination revealed nothing, though the blood was searched for signs of phlebitis, examined for crystals, and for the flakes of pigment described by Brown Séquard. As it has been found by Ludwig and Haffer, that section of the splanchnic nerves will kill animals in one or two days, and according to Brown Séquard, mere pricking or cutting of the semi-lunar ganglion proves fatal to rabbits in thirty hours, while division of the sympathetic in the neighborhood of the kidneys causes death in twenty-three hours: in absence of other proof of death, it is probable that it resulted from injury done, in tearing out the capsules, to the ganglionic system of nerves.

The extirpation of the right is more fatal than the removal of the left capsule, as is proved by experiments which the author has cited, but which it is unnecessary for us to state. M. Gratiolet thought that this fact was to be explained by the proximity of the liver, and the occurrence of hepatitis; at first Dr. Harley was of this opinion, but he now very justly concludes that it is due to another cause. The right semi-lunar ganglion, which is much larger than the left, lies (in the dog and cat,) directly beneath the supra-renal capsule; while the smaller left semi-lunar ganglion is placed on the left crus of the diaphragm, and internal to the supra-renal body. Thus it will be immediately seen that a removal of the right capsule will do more mischief to this ganglion, and this injury has been shown by Ludwig, Haffer and Brown Séquard to be sufficient to produce death in a short time.

As it is generally the case in the removal of double organs, that if one be left it becomes hypetrophied from being compelled to perform a double function. Dr. Harley was anxious to observe if this was the case as respects the supra-renal capsutles. He accordingly removed the right capsule from a cat, which had made an excellent recovery from the operation of removing the left about a month before. Nothing unusual, however, was observed: on the morning of the third day it died, and on opening the abdomen, two of the lumbar lymphatic glands were observed very much enlarged, and presenting a peculiarly beautiful, semi-transparent, lobular appearance.

Dr. Harley performed another experiment on a piebald rat, with reference to the same point. . The right supra-renal capsule was removed, and the animal made a rapid recovery, being in excellent condition at the end of six weeks. The left capsule was then removed, and compared with others taken from animals of the same species and age, in order to ascertain if it had become hypertrophied. But in this, as in the case of the cat, there was no very marked difference in either its size or appearance. From the effects of the latter operation the animal speedily recovered, and ultimately became very fat and healthy looking.

An experiment was made on a white rat, in order to observe if any change would take place in the color of the hair and skin. Both capsules were removed at an interval of nine days. The animal lived sixteen days after the last operation, and during that time the color of the hair and skin was carefully watched, without discovering any alteration, excepting that the neck became denuded of hair, was covered with a luxuriant crop of young hair, about an eighth of an inch long.

The foregoing experiments, which we have presented in as condensed a form as was consistent, cannot fail to strike every one as extremely interesting, and leading to some very important conclusions. Brown Séquard has maintained that it is impossible for an animal to live for more than forty-eight hours at the farthest, after it has been deprived of its supra-renal capsules. The experiments of Dr. Harley fully controvert this statement, and in addition, M. Philipeaux, of Paris, has removed the spleen, supra-renal capsules, and the thyroid body from the same animal, which afterwards recovered. Dr. Harley brought with him from Paris a young rat, the offspring of a mother which had been deprived of spleen and supra-renal capsules Brown Séquard is also probably in the wrong in supposing that the convulsions and turning which followed his experiments, were due to the removal of the supra-renal capsules; as Dr. Harley has not observed it in his experiments, and is confirmed by a private letter from Professor Virchow, who has also failed to notice this phenomenon.

We had the pleasure, from the politeness of Dr. Brown Séquard, of examining certain microscopic crystals, which he invariably found in the blood of animals from which he had removed the supra-renal capsules. These crystals were unlike the ordinary blood crystals, being pale and needle shaped. Dr. Harley makes no allusion to this occurrence.

The article, of which we have given a *resumé*, is not yet concluded, but will be continued in the next number of the British and Foreign Review: it seemed, however, to have treated of certain points with sufficient completeness to make an analysis of it, even now, profitable to our readers; when it is concluded, we will continue our analysis in another number of the Journal. We ourselves had an opportunity of seeing a case, which was probably an example of Addison's disease, before we knew of Addison's monograph; and though they are extremely rare, yet they are liable to occur in the practice of any one; we would earnestly request, that if any of our readers should meet with such a case, that they will send us its history, and if fatal, a minute account of the appearances of the supra-renal capsules. It is in this way only that we can make progress in understanding this new disease, as it is not likely that a sufficient number of cases will come under the observation of a single individual for purposes of analysis.

Finally, we present the conclusions drawn by Dr. Harley from the results of his experiments.

1st. *The supra-renal capsules are not solely fœtal organs.*

2d. *The supra-renal capsules are not absolutely essential to life.*

3d. *The removal of the right is generally more fatal than removal of the left capsule.*

4th. *That convulsions do not necessarily follow the removal of the capsule.*

5th. *That the absence of this function (in rats,) is attended neither by great emaciation nor debility.*

6th. *That when death follows upon the extirpation of the supra-renal bodies, it is in most cases in consequence of the injury done to the neighboring tissues; perhaps most frequently the mutilation of the ganglionic system of nerves.*

7th. *Absence of the function of the supra-renal bodies is not proved to have any special effect in arresting the transformation of hœmatin, or in increasing the formation of blood crystals.*

8th. *The suppression of the supra-renal capsular function is not attended by any increased deposit of pigment in the skin in its appendages (in rats.)*

9th. *The problem of the connection of bronzed skin and supra-renal capsular disease is more likely to be solved in the dead house than in the physiological laboratory.* F.

